# Prevalence and correlates of periodontitis among Kenyan women planning to conceive

**DOI:** 10.1186/s12903-022-02243-w

**Published:** 2022-05-31

**Authors:** Brenda Oyaro, Erica Lokken, Hudson Alumera, Shahid Hussein, Barbra Richardson, Kishorchandra Mandaliya, Walter Jaoko, John Kinuthia, Elizabeth Dimba, Arthur Kemoli, R. Scott McClelland

**Affiliations:** 1 Department of Medicine, University of Washington, 325 9th Avenue, Box 359909, Seattle, WA 98104 USA; 2 Department of Medical Microbiology, University of Nairobi, Nairobi, Kenya; 3 Department of Global Health, University of Washington, Seattle, WA USA; 4 Department of Periodontology, Community and Preventive Dentistry, School of Dental Sciences, University of Nairobi, Nairobi, Kenya; 5 Coast General Teaching and Referral Hospital, Mombasa, Kenya; 6 Department of Biostatistics, University of Washington, Seattle, WA USA; 7 PathCare Laboratories, Mombasa, Kenya; 8 Department of Paediatric Dentistry and Orthodontics, School of Dental Sciences, University of Nairobi, Nairobi, Kenya; 9 Department of Oral Pathology, School of Dental Sciences, University of Nairobi, Nairobi, Kenya; 10 Kenyatta National Hospital, Nairobi, Kenya; 11 Department of Epidemiology, University of Washington, Seattle, WA USA

**Keywords:** Periodontitis, Gingivitis, DMFT, Women, Kenya

## Abstract

**Background:**

Periodontitis has been associated with adverse pregnancy outcomes. Little is known about the burden and risk factors for periodontitis among reproductive age women in sub-Saharan Africa. This analysis aimed to determine the prevalence and correlates of periodontitis among Kenyan women planning to conceive.

**Methods:**

HIV-seronegative, reproductive-age women who were planning to conceive were enrolled and underwent a periodontal examination. Following the US Centers for Disease Control and Prevention clinical case definitions, the presence and severity of periodontitis was determined by establishing the level of clinical periodontal attachment loss and graded in three categories: no/mild, moderate, and severe. Secondary outcomes included the scores on the Gingival Index and Decayed, Missing, and Filled Teeth (DMFT) Index. Correlates of periodontitis were examined using univariable and multivariable logistic regression.

**Results:**

Of the 647 women in the study, 84% (n = 541) had no/mild periodontitis, 15% (n = 97) had moderate periodontitis, and 1% (n = 9) had severe periodontitis. Mild gingivitis was present in 61% (n = 396) of women, while 27% (n = 176) had moderate gingivitis, and 1% (n = 9) had severe gingivitis. The majority (75%, n = 487) of women had a DMFT index in the very low range (score < 5). Periodontitis was observed in 12% (12/101) of nulliparous women compared to 13% (36/286) of women with one prior delivery (prevalence ratio [PR] 1.03, 95% confidence interval [95% CI] 0.57–1.96), 21% (36/170) of women with two prior deliveries (PR 1.78, 95% CI 0.97–3.26), and 24% (22/90) of women with 3 or more prior deliveries (PR 2.06, 95% CI 1.08–3.92).

**Conclusion:**

This study demonstrated a substantial prevalence of moderate-severe periodontitis among women planning to conceive in Kenya. These results highlight the need to address the oral care needs of reproductive age women, particularly those with multiple prior pregnancies.

## Introduction

Periodontitis is among the commonest diseases of the oral cavity, with an estimated global prevalence of 27% [1]. It is a chronic inflammatory condition caused by microbial infection of the supporting tissues around the teeth that include the gingiva, alveolar bone, and periodontal ligament. Clinical diagnosis of periodontitis is based on measures of periodontal pocket depth, interdental clinical attachment loss, pattern and extent of alveolar bone loss, or a combination of these measures [2].

Risk factors associated with periodontitis include poor oral hygiene, cigarette smoking, parity, and hormonal changes in women especially during pregnancy [3–6]. Inflammatory mediators including the NOD-like receptor pyrin domain-containing 3 (NLRP3) and enzymes like transglutaminase may play a role in the pathogenesis and progression of periodontitis [7, 8]. Additionally, periodontitis has been associated with systemic illnesses like cardiovascular disease and rheumatoid arthritis as well as adverse pregnancy outcomes, including preeclampsia and preterm birth [9]. Periodontitis is often studied in pregnant women because of its association with adverse pregnancy outcomes [10]. One recognized limitation of assessing this disease during pregnancy is the fact that hormonal changes during pregnancy lead to changes in periodontal tissues, increasing the likelihood of diagnosing periodontitis during this period [6].

Few studies have examined periodontal disease status among women planning for a pregnancy. The rationale for conducting this analysis was to understand the prevalence and risk factors associated with periodontitis in reproductive-age women planning pregnancy. This is an important population for further study, as intervention to address periodontitis prior to conception holds potential for reducing the risk of periodontitis-associated adverse pregnancy outcomes.

## Methods

### Study population

This cross-sectional analysis used data collected during the enrollment and periodontal examination visits in the Microbiota and Preterm Birth study [11]. The primary aim of the parent study was to test the hypothesis that the vaginal microbiota present near to the time of conception is associated with women’s risk of spontaneous preterm birth. The study included reproductive age HIV-seronegative women with immediate fertility intent. Additional eligibility criteria included being ≤ 45 years old, planning to become pregnant and remain in the study area through pregnancy, having a menstrual period in the prior 3 months or recently discontinued contraceptive methods that induce amenorrhea (e.g., implant, hormonal intrauterine device), willing to comply with study procedures, and able to provide informed consent. Women were excluded if they were currently pregnant, using contraception other than condoms for STI prevention, had a history of uterine or cervical surgery other than caesarean section, or reported a history of seeking treatment for infertility. Recruitment was conducted through several sources including family planning clinics, the Discordant Couples Clinic (DCC) at Kenyatta National Hospital (KNH), and referrals from study participants. All eligible women referred from any of these sources were invited to participate in the study after completing the informed consent process.

### Sample size determination

The sample size for this analysis was driven by the total number of participants enrolled in the parent study (N = 701) [11].

### Study procedures

At enrollment, a standardized face-to-face interview was conducted by study clinicians/nurses to collect information on sociodemographic characteristics; sexual behavior; reproductive, contraceptive, and medical history; cigarette smoking; and alcohol use using the Alcohol Use Disorders Identification Test (AUDIT) [12]. A physical examination was performed by an experienced study clinician, including height and weight measurements that were used to calculate body mass index (BMI).

Periodontal examination visits were scheduled within a month of the enrollment visit. Initially, a brief interview to collect information on oral hygiene and health seeking behavior was done followed by periodontal examination conducted by experienced co-investigators in their respective clinics at the University of Nairobi Dental School (HA) and the Coast General Teaching and Referral Hospital Dental Unit (SH). Urgent periodontal care needs including scaling, radiographs, and filling/extraction were addressed at no cost to the participant.

The presence and severity of periodontitis was determined by establishing the level of clinical attachment loss (CAL). This was calculated as the sum of the clinical attachment measurement and the probing pocket depth. Clinical attachment measurement, which is the distance between the cement-enamel junction (CEJ) and the gingival margin, was recorded to the nearest whole millimeter using a periodontal probe (University of Michigan “O” probe with William’s markings, Hu-Friedy Co. USA). This variable was given a positive value when there was gingival recession and a negative value when the gingival margin was coronal to the CEJ. Pocket measurement was done on the following teeth—16, 12, 24, 36, 32 and 44 according to Ramfjord’s index [13]. The level of periodontitis was categorized as no/mild, moderate, or severe based on the clinical attachment loss values. Severe periodontitis was defined based on the presence of ≥ 2 interproximal sites with CAL ≥ 6 mm (not on same tooth). Moderate periodontitis was defined based on the presence of ≥ 2 interproximal sites with CAL ≥ 4 mm but < 6 mm (not on same tooth). Individuals not meeting criteria for moderate or severe periodontitis were classified as having no or mild periodontitis.

The Gingival Index, a measure for assessing severity of gingivitis, was determined by assessing the degree of gingival inflammation and categorized as normal gingiva, mild inflammation (slight change in color, slight edema, no bleeding on probing), moderate inflammation (redness, edema, glazing, bleeding on probing), or severe inflammation (marked redness, edema, ulceration, prone to spontaneous bleeding) [14]. The Decayed, Missing, and Filled Teeth (DMFT) Index was calculated as the sum of the number of decayed, missing, and filled teeth due to dental caries. This was subsequently categorized as very low < 5, low 5–8.9, moderate 9–13.9 and high ≥ 14 [15].

Calibrations were conducted at least three times per year. At the Nairobi site, an experienced periodontist served as the gold standard examiner for (HA), who then served as the gold standard examiner for (SH), the dentist in Mombasa. Both the primary examiner and the gold standard examiner conducted parallel examinations on five individuals. Kappa scores were calculated to characterize the level of agreement between the two scores. A score of 0.8 was the pre-determined inter-examiner level of agreement and lower levels were addressed through discussion and agreement between the gold standard examiner and the examiner at the site. The results for the inter-examiner calibration were 0.86 and 0.84 for Nairobi and Mombasa respectively. Intra-examiner variability was evaluated after every 50 participants at each study site, with results being kappa 1.00 for both Nairobi and Mombasa. During the intra-examiner variability calibration, one participant underwent the periodontal exam twice and results of the two periodontal exams were compared across both attempts.

### Data analysis

Baseline characteristics were summarized as median and interquartile range (IQR) for continuous variables and count and percentage for categorical variables. Some categories of baseline characteristics were merged based on scientific or statistical considerations. Body mass index data were analyzed as a binary outcome with obese and non-obese categories using the standard obese cut-off of ≥ 30 to define obesity. These categories were chosen because obesity has been identified as a risk factor for periodontitis [16, 17], while no differences in risk have been observed across the lower categories of BMI. Household income categories were collapsed into three categories for analysis, as there were very few observations in the < Ksh 2500 and > Ksh 75,000 income categories. The primary outcome was periodontitis, classified as no/mild, moderate, and severe following the clinical case definitions proposed by the CDC working group for use in population-based surveillance [2]. The severe periodontitis outcome category contained very few positive observations, so it was combined with the moderate periodontitis category for analysis. Similar combined categories were created for categories of the Gingival and DMFT indices. Univariate logistic regression was used to estimate the association between each correlate and the presence of moderate or severe periodontitis. Variables associated with moderate or severe periodontitis in univariate analysis (p < 0.1) were included in the multivariable model. The level of significance used to select variables for inclusion in the multivariable model was not based on statistical significance at the alpha = 0.05 level. A higher p-value threshold was used because variables do not need to be statistically significant to be confounders of other variables in a multivariable model. All associations were reported as prevalence ratios (PR) with 95% confidence intervals (95% CI). Similar analyses were conducted for the secondary outcomes (Gingival Index and DMFT Index).

## Results

Between April 2017 and March 2020, 701 women were enrolled, of whom 92% (n = 647) had a periodontal examination. Baseline characteristics of the 647 women are presented in Table 1. Of the 647 women enrolled, 65%, (n = 423) were from Nairobi and 35%, (n = 224) from Mombasa. Their median age was 29 (IQR 25–34) years, and most (96%, n = 622) were either married or living with a partner. Only 0.5% (n = 3) women were current smokers, and 2% (n = 14) were classified as having hazardous or harmful alcohol use (AUDIT score ≥ 7). A quarter of the women (25%, n = 156) were obese based on BMI ≥ 30. Nearly all participants (99%, n = 643) reported cleaning their teeth with toothbrushes, with 94% (n = 608) of women reporting using fluoride-containing toothpaste. Almost half (47%, n = 303) had received no previous dental care. On examination, 84% (n = 541) of the women had no/mild periodontitis, 15% (n = 97) had moderate periodontitis, and only 1% (n = 9) had severe periodontitis. Ten percent (n = 65) of the women had no gingivitis, 61% (n = 396) had mild gingivitis, 27% (n = 176) had moderate gingivitis, and only 1% (n = 9) had severe gingivitis. The majority (75%, n = 487) of women had a very low (< 5) DMFT Index, while 16% (n = 101) had a low (5–8.9) DMFT Index, 7% (n = 44) had a moderate (9–13.9) DMFT Index, and 2% (n = 14) had a high (≥ 14) DMFT Index.

**Table 1:** Baseline characteristics of 647 Kenyan women planning for pregnancy

Characteristic	Median (IQR) or N (%)
Age (years)	29 (24–34)
*Level of education (years)*	
0–8	164 (25.3)
9–12	250 (38.6)
> 12	253 (36.0)
*Study site*	
Nairobi	423 (65.4)
Mombasa	224 (34.6)
*Marital status*	
Married/living with partner	622 (96.1)
*Monthly household income (Ksh)*	
≤ 2,500–10,000	225 (35.0)
10,001–30,000	277 (43.0)
≥ 30,001	141 (21.9)
Availability of household water and electricity	416 (64.3)
Current smoker	3 (0.5)
*Alcohol use (AUDIT score)*	
0 (non-drinker)	560 (86.6)
1–6 (Non-hazardous)	73 (11.3)
≥ 7 (Hazardous)	14 (2.2)
Chronic illness*	22 (3.2)
Frequency of teeth cleaning/week	6 (5.6)
Toothbrush use	643 (99.4)
Fluoride-containing toothpaste use	132 (38.2)
*Time since last dental visit*	
< 6 months	33 (5.1)
6–12 months	61 (9.4)
> 1 year but < 2 years	39 (6.0)
≥ 2 years but < 5 years	89 (13.8)
≥ 5 years	122 (18.9)
Never received dental care	303 (46.8)
Obese (BMI ≥ 30)	156 (24.5)
*Parity*	
Nulliparous	101 (15.6)
1	286 (44.2)
2	170 (26.3)
≥ 3	90 (13.9)
*Gravidity*	
0	62 (9.6)
≥ 1	585 (90.4)

*Women reporting history of any chronic illness including hypertension and diabetes mellitus

Correlates of moderate-severe periodontitis are presented in Table 2; only parity, age, and education were associated at p < 0.1. Periodontitis was observed in 12% (12/101) of nulliparous women compared to 13% (36/286) of women with one prior delivery (PR 1.03, 95% CI 0.57–1.96), 21% (36/170) of women with two prior deliveries (PR 1.78, 95% CI 0.97–3.26), and 24% (22/90) of women with 3 or more prior deliveries (PR 2.06, 95% CI 1.08–3.92). There was a statistical trend for increased prevalence of moderate-severe periodontitis with increasing age (Wald p-value = 0.1). The prevalence of moderate-severe periodontitis in women < 25 years old was 12% (16/135), while moderate-severe periodontitis was observed in 15% (30/207) of women aged 25–29 years (PR 1.22, 95% CI 0.69–2.16), 17% (27/161) women aged 30–34 years (PR 1.42, 95% CI 0.80–2.51), 23% (26/115) of women aged 35–39 years (PR 1.91, 95% CI 1.08–3.38), and 24% (7/29) of women > 40 years old (PR 2.04, 95% CI 0.92–4.50). There was also a trend for lower prevalence of periodontitis with increasing educational level (Wald p-value = 0.1). The prevalence of moderate-severe periodontitis in women with 0–8 years of education was 20% (33/164), while moderate-severe periodontitis was observed in 18% (44/250) of women with 9–12 years of education (PR 0.88, 95% CI 0.58–1.31) and 12% (29/233) of women with > 12 years of education (PR 0.62, 95% CI 0.39–0.98). Age and educational level were collinear, so only educational level was included in the multivariable model, as this variable was more strongly associated with moderate-severe periodontitis in univariable analysis. In the multivariable analysis including educational level, the association between parity and moderate-severe periodontitis was similar to univariable results.

**Table 2:** Correlates of moderate-severe periodontitis in Kenyan women planning for pregnancy

Characteristic	No/Mild Periodontitis n = 541 N (%)	Moderate-severe periodontitis n = 106 N (%)	Unadjusted PR (95% CI)	p value	Adjusted PR (95% CI)	p value
*Study site*						
Nairobi	353 (83.5)	70 (16.5)	1.0	0.9		
Mombasa	187 (83.9)	36 (16.1)	0.98 (0.68–1.41)			
*Age, years*						
< 25	119 (88.1)	16 (11.9)	1.0	0.1		
25–29	177 (85.5)	30 (14.5)	1.22 (0.69–2.16)			
30–34	134 (83.2)	27 (16.8)	1.42 (0.80–2.51)			
35–39	89 (77.4)	26 (22.6)	1.91 (1.08–3.38)			
> 40	22 (75.9)	7 (24.1)	2.04 (0.92–4.50)			
*Highest education level*^*a*^						
0–8 years	131 (79.9)	33 (20.1)	1.0	0.1	1.0	0.2
9–12 years	206 (82.4)	44 (17.6)	0.88 (0.58–1.31)		1.01 (0.67–1.52)	
> 12 years	204 (87.6)	29 (12.4)	0.62 (0.39–0.98)		0.69 (0.44–1.10)	
*Socioeconomic status (monthly earnings in Ksh)*						
≤ 10,000	181 (80.4)	44 (19.6)	1.0	0.2		
10,001–30,000	234 (84.5)	43 (15.5)	0.79 (0.54–1.16)			
≥ 30,001	123 (87.2)	18 (12.8)	0.65 (0.39–1.08)			
*Availability of household water and electricity*						
No	189 (81.8)	42 (18.2)	1.0	0.4		
Yes	352 (84.6)	64 (15.4)	0.85 (0.59–1.21)			
*Alcohol use (AUDIT score)*						
0 (non-drinker)	465 (83.0)	95 (17.0)	1.0	0.4		
1–6 (Non-hazardous)	65 (89.0)	8 (11.0)	0.65 (0.33–1.27)			
≥ 7 (Hazardous)	11 (78.6)	3 (21.4)	1.26 (0.46–3.50)			
*Chronic illness*						
No	524 (83.8)	101 (16.2)	1.0	0.4		
Yes	17 (77.3)	5 (22.7)	1.41 (0.64–3.10)			
*Frequency of teeth cleaning per day*						
< 2	183 (82.1)	40 (17.9)	1.0	0.4		
≥ 2	358 (84.4)	66 (15.6)	0.87 (0.61–1.24)			
*Time period since last dental visit*						
0–12 months	86 (91.5)	8 (8.5)	1.0	0.3		
> 1 year but < 2 years	32 (82.1)	7 (17.9)	2.11 (0.82–5.42)			
≥ 2 years but < 5 years	72 (80.9)	17 (19.1)	2.24 (1.02–4.94)			
≥ 5 years	99 (81.1)	23 (18.9)	2.22 (1.04–4.73)			
Never received dental care	252 (83.2)	51 (16.8)	1.98 (0.97–4.02)			
*BMI*						
Non-obese < 30	409 (84.9)	73 (15.1)	1.0	0.2		
Obese ≥ 30	125 (80.1)	31 (19.9)	1.31 (0.90–1.92)			
*Parity*^*b*^						
Nulliparous	89 (88.1)	12 (11.9)	1.0	0.01	1.0	0.02
1	250 (87.4)	36 (12.6)	1.03 (0.57–1.96)		1.06 (0.57–1.95)	
2	134 (78.8)	36 (21.2)	1.78 (0.97–3.26)		1.77 (0.97–3.25)	
≥ 3	68 (75.6)	22 (24.4)	2.06 (1.08–3.92)		1.99 (1.04–3.82)	
*Gravidity*^*c*^						
0	55 (88.7)	7 (11.3)	1.0	0.3		
≥ 1	486 (83.1)	99 (16.9)	1.50 (0.73–3.08)			

^a^Included in multivariable model

^b^Number of pregnancies that reached viability (> 20 weeks’ gestation)

^c^Total number of pregnancies including those ending in abortion, miscarriage, and ectopic pregnancy

Correlates of gingivitis are presented in Table 3. In univariable analyses, moderate-severe gingivitis was more common in women from Mombasa compared to Nairobi. Lower prevalences were observed in association with increasing levels of education and monthly household income. In a multivariable analysis including all three variables, the association with site was similar, while the associations with education and income were attenuated, and no longer statistically significant.

**Table 3:** Correlates of moderate-severe gingivitis in Kenyan women planning for pregnancy

Characteristic	No/Mild gingivitis n = 461 N (%)	Moderate-severe gingivitis n = 185 N (%)	Unadjusted PR (95% CI)	p value	Adjusted PR (95% CI)	p value
*Study site*^*a*^						
Nairobi	315 (74.6)	107 (25.4)	1.0	0.01	1.0	0.04
Mombasa	146 (65.2)	78 (34.8)	1.37 (1.08–1.75)		1.30 (1.01–1.66)	
*Age, years*						
< 25	97 (71.9)	38 (28.1)	1.0	0.9		
25–29	152 (73.4)	55 (26.6)	0.94 (0.66–1.34)			
30–34	113 (70.2)	48 (29.8)	1.06 (0.74–1.52)			
35–39	79 (69.3)	35 (30.7)	1.09 (0.74–1.60)			
> 40	20 (69.0)	9 (31.0)	1.10 (0.60–2.02)			
*Highest education level*^*a*^						
0–8 years	102 (62.2)	62 (37.8)	1.0	0.002	1.0	0.07
9–12 years	176 (70.7)	73 (29.3)	0.78 (0.59–1.02)		0.82 (0.62–1.08)	
> 12 years	183 (78.5)	50 (21.5)	0.57 (0.41–0.78)		0.65 (0.45–0.94)	
*Socioeconomic status*^*a*^* (monthly earnings in Ksh)*						
≤ 10,000	144 (64.3)	80 (35.7)	1.0	0.01	1.0	0.2
10,001–30,000	206 (74.4)	71 (25.6)	0.72 (0.55–0.94)		0.78 (0.59–1.02)	
≥ 30,001	108 (76.6)	33 (23.4)	0.66 (0.46–0.93)		0.85 (0.60–1.26)	
*Availability of household water and electricity*						
No	157 (68.3)	73 (31.7)	1.0	0.2		
Yes	304 (73.1)	112 (26.9)	0.85 (0.66–1.09)			
*Alcohol use (AUDIT score)*						
0 (non-drinker)	394 (70.5)	165 (29.5)	1.0	0.4		
1–6 (Non-hazardous)	56 (76.7)	17 (23.3)	0.79 (0.51–1.22)			
≥ 7 (Hazardous)	11 (78.6)	3 (21.4)	0.73 (0.26–2.00)			
*Chronic illness*						
No	445 (71.3)	179 (28.7)	1.0	0.9		
Yes	16 (72.7)	6 (27.3)	0.95 (0.48–1.90)			
*Frequency of teeth cleaning per day*						
< 2	158 (712)	64 (28.8)	1.0	0.9		
≥ 2	303 (71.5)	121 (28.5)	0.99 (0.77–1.28)			
*Time period since last dental visit*						
0–12 months	72 (77.4)	21 (22.6)	1.0	0.4		
> 1 year but < 2 years	30 (76.9)	9 (23.1)	1.02 (0.52–2.03)			
≥ 2 years but < 5 years	58 (65.2)	31 (34.8)	1.54 (0.96–2.47)			
≥ 5 years	88 (72.1)	34 (27.9)	1.23 (0.77–1.98)			
Never received dental care	213 (70.3)	90 (29.7)	1.32 (0.87–1.99)			
*BMI*						
Non-obese < 30	344 (71.5)	137 (28.5)	1.0	0.9		
Obese ≥ 30	112 (71.8)	44 (28.2)	0.99 (0.74–1.32)			
*Parity*						
Nulliparous	73 (72.3)	28 (27.7)	1.0	0.9		
1	206 (72.3)	79 (27.7)	1.00 (0.69–1.44)			
2	120 (70.6)	50 (29.4)	1.06 (0.72–1.57)			
≥ 3	62 (68.9)	28 (31.1)	1.12 (0.72–1.74)			
*Gravidity*						
0	47 (75.8)	15 (24.2)	1.0	0.4		
≥ 1	414 (70.9)	170 (29.1)	1.20 (0.76–1.90)			

^a^Included in multivariable model

Correlates of DMFT index are presented in Table 4. In univariable analyses, higher prevalences of DMFT index in the upper range (≥ 5) were observed in association with increasing age and gravidity. The prevalence of DMFT index in the upper range (≥ 5) was significantly lower in women who had never received dental care. Age and time period since last dental visit were collinear, hence only age was included in the multivariable model since it was more strongly associated with the outcome. Results were similar in a multivariable model that included age and gravidity.

**Table 4:** Correlates of DMFT index in Kenyan women planning for pregnancy

Characteristic	Very low DMFT index n = 487 N (%)	Low/Mod/High DMFT index n = 159 N (%)	Unadjusted PR (95% CI)	p value	Adjusted PR (95% CI)	p value
*Study site*						
Nairobi	314 (74.2)	109 (25.8)	1.0	0.4		
Mombasa	173 (77.6)	50 (22.4)	0.87 (0.65–1.17)			
*Age, years*^*a*^						
< 25	109 (80.7)	26 (19.3)	1.0	0.02	1.0	0.04
25–29	166 (80.2)	41 (19.8)	1.03 (0.66–1.60)		0.97 (0.62–1.50)	
30–34	116 (72.5)	44 (27.5)	1.43 (0.93–2.19)		1.31 (0.85–2.02)	
35–39	75 (65.2)	40 (34.8)	1.81 (1.18–2.77)		1.66 (1.08–2.55)	
> 40	21 (72.4)	8 (27.6)	1.43 (0.72–2.84)		1.29 (0.65–2.56)	
*Highest education level*						
0–8 years	126 (76.8)	38 (23.2)	1.0	0.8		
9–12 years	185 (74.3)	64 (25.7)	1.11 (0.78–1.57)			
> 12 years	176 (75.5)	57 (24.5)	1.06 (0.74–1.51)			
*Socioeconomic status (monthly earnings in Ksh)*						
≤ 10,000	174 (77.3)	51 (22.7)	1.0	0.7		
10,001–30,000	205 (74.0)	72 (26.0)	1.15 (0.84–1.57)			
≥ 30,001	105 (75.0)	35 (25.0)	1.10 (0.76–1.61)			
*Availability of household water and electricity*						
No	175 (75.8)	56 (24.2)	1.0	0.9		
Yes	312 (75.2)	103 (24.8)	1.02 (0.77–1.36)			
*Alcohol use (AUDIT score)*						
0 (non-drinker)	418 (74.8)	141 (25.2)	1.0	0.7		
1–6 (Non-hazardous)	58 (79.5)	15 (20.5)	0.82 (0.51–1.31)			
≥ 7 (Hazardous)	11 (78.6)	3 (21.4)	0.85 (0.31–2.34)			
*Chronic illness*						
No	469 (75.2)	155 (24.8)	1.0	0.5		
Yes	18 (81.8)	4 (18.2)	0.73 (0.30–1.80)			
*Frequency of teeth cleaning per day*						
< 2	169 (75.8)	54 (24.2)	1.0	0.9		
≥ 2	318 (75.2)	105 (24.8)	1.03 (0.77–1.36)			
*Time period since last dental visit*^*a*^						
0–12 months	61 (64.9)	33 (35.1)	1.0	< 0.001		
> 1 year but < 2 years	25 (64.1)	14 (35.9)	1.02 (0.62–1.69)			
≥ 2 years but < 5 years	56 (62.9)	33 (37.1)	1.06 (0.72–1.55)			
≥ 5 years	81 (66.9)	40 (33.1)	0.94 (0.65–1.37)			
Never received dental care	264 (87.1)	39 (12.9)	0.37 (0.25–0.55)			
*BMI*						
Non-obese < 30	366 (76.1)	115 (23.9)	1.0	0.5		
Obese ≥ 30	115 (73.7)	41 (26.3)	1.10 (0.81–1.50)			
*Parity*						
Nulliparous	83 (82.2)	18 (17.8)	1.0	0.4		
1	214 (75.1)	71 (24.9)	1.40 (0.88–2.23)			
2	125 (73.5)	45 (26.5)	1.49 (0.91–2.42)			
≥ 3	65 (72.2)	25 (27.8)	1.56 (0.91–2.66)			
*Gravidity*^*b*^						
0	54 (87.1)	8 (12.9)	1.0	0.02	1.0	0.09
≥ 1	433 (74.1)	151 (25.9)	2.00 (1.04–3.88)		1.79 (0.91–3.50)	

^a^Age and time-period since last dental visit were collinear, so only age was included in the multivariable model

^b^Included in multivariable model

## Discussion

In this population of Kenyan women planning to conceive, nearly one out of six had moderate-severe periodontitis, and the prevalence of moderate-severe periodontitis was almost twice as high in multiparous women compared to those with parity of one or less. The prevalence of moderate-severe gingivitis, a precursor to periodontitis was 29%.

Studies in different settings have shown wide variation in the prevalence of periodontitis. In a US-based study using combined data from the 2009–2010 and 2011–2012 cycles of the National Health and Nutrition Examination Survey, the prevalence of periodontitis was estimated at 46%, with 8.9% of the population having severe periodontitis [18]. In a similar survey conducted among pre-conception women at the Maternal and Child Health Hospital, Changzhou, China between January 2012 and December 2014, the overall prevalence of periodontitis was 74%. In this Chinese cohort, 22% had mild periodontitis, 51% had moderate periodontitis and 1% had severe periodontitis [19]. A cross-sectional study in a Tanzanian cohort of pregnant women found a 5% prevalence of severe periodontitis [20]. Differences in the prevalence of periodontitis across these different studies may be attributed to differences in study design, definitions of periodontitis, and differences in the age, race, access to dental care, and socioeconomic status of study populations [1, 21, 22].

The association between higher parity and increased likelihood of periodontitis observed in this population of Kenyan women is consistent with findings from other studies [23–25]. An important biological mechanism explaining the higher risk of periodontitis with increasing parity is that pregnancy causes a rise in circulating levels of estrogen and progesterone. Human gingiva contains receptors for estrogen and progesterone, and increased plasma levels result in accumulation of these hormones in gingival tissues causing changes in sub-gingival microbiota, increased vascular permeability, and greater susceptibility to inflammation [6, 26]. While the inflammation of periodontal tissues during pregnancy is temporary [27], destruction of periodontal tissue may persist even after childbirth. Repeated occurrences of untreated periodontitis during multiple pregnancies may explain the association between higher parity and the presence of moderate-severe periodontitis [28]. The observed association between periodontitis and higher parity could also be a result of confounding by other factors such as age. Multiparous women tend to be older than nulliparous or uniparous women, and periodontal disease prevalence increases with age [5]. However, this and other studies have observed that the association is not eliminated in analyses adjusting for age, suggesting that the effect of parity on periodontal health is not due to age alone. Inequities in access to healthcare among women of lower socioeconomic status and lower educational levels, who also tend to have more children, could also contribute to the observed association [29]. Nonetheless, sociodemographic variables and educational level were only modestly associated with moderate-severe periodontitis in this Kenyan cohort, and adjustment for educational attainment did not attenuate the association between parity and periodontal disease.

Several studies and systematic reviews have concluded that there is an association between periodontitis and spontaneous preterm birth [30–33]. Despite this consistent association, clinical trials have generally not found that treatment of periodontal disease reduces the risk for adverse pregnancy outcomes. Several possible explanations have been proposed including lack of a causal association, shared risk factors like socioeconomic status and smoking, insufficiently powered clinical trials, variable definitions of periodontitis, and treatment that may have been too late or insufficiently aggressive to improve pregnancy outcomes [34]. Populations like this cohort of Kenyan women would be ideal for future longitudinal studies to determine whether earlier, more aggressive, and more prolonged interventions addressing periodontitis could improve birth outcomes.

A unique finding of this analysis was that never receiving dental care was protective against a high DMFT index. The explanation could be that women who had not experienced problems with tooth decay never sought dental care. Further these women were younger, and might have had access to preventive oral health care information, making them less likely to have had oral problems that would require dental care.

This study had several strengths. First, women were examined prior to becoming pregnant, enabling preconception exposure measurement. Additionally, standardized periodontal examinations were conducted by experienced clinicians with rigorous quality control. The population size (N = 647) also provided sufficient power for this analysis.

The findings from this analysis should be interpreted in the context of several limitations. First, the cross-sectional design does not provide evidence of a temporal relationship between some exposures and outcomes. Second, this analysis included multiple comparisons, increasing the risk that observed associations might be due to chance. Nonetheless, the association between multi-parity and periodontitis identified in this population was consistent with findings from prior studies [23–25, 35].

## Conclusion

This study demonstrated a substantial prevalence of moderate-severe periodontitis among Kenyan women planning to conceive. These findings highlight the need to address the oral health care needs of reproductive age women, especially those who are multiparous, since the prevalence of moderate-severe periodontitis was particularly high in this group. Advances in the field of salivary and periodontal tissue biomarkers as potential therapeutic targets for treatment and prevention of oral inflammatory and immunological diseases could lead to early diagnosis and treatment of oral inflammatory conditions like periodontitis. Questions remain about whether earlier and more aggressive treatment of periodontal disease could improve pregnancy outcomes in populations like this one.

## Data Availability

The authors confirm that all relevant data are within the paper.

## Abbreviations

AUDIT: Alcohol Use Disorders Identification Test
BMI: Body Mass Index
CAL: Clinical Attachment Loss
CEJ: Cement Enamel Junction
CI: Confidence Interval
DCC: Discordant Couples Clinic
DMFT: Decayed, Missing, and Filled Teeth
IQR: Inter-quartile range
KNH: Kenyatta National Hospital
NICHD: National Institute Child Health and Human Development
NIH: National Institutes of Health
PR: Prevalence Ratio
STI: Sexually Transmitted Infection
